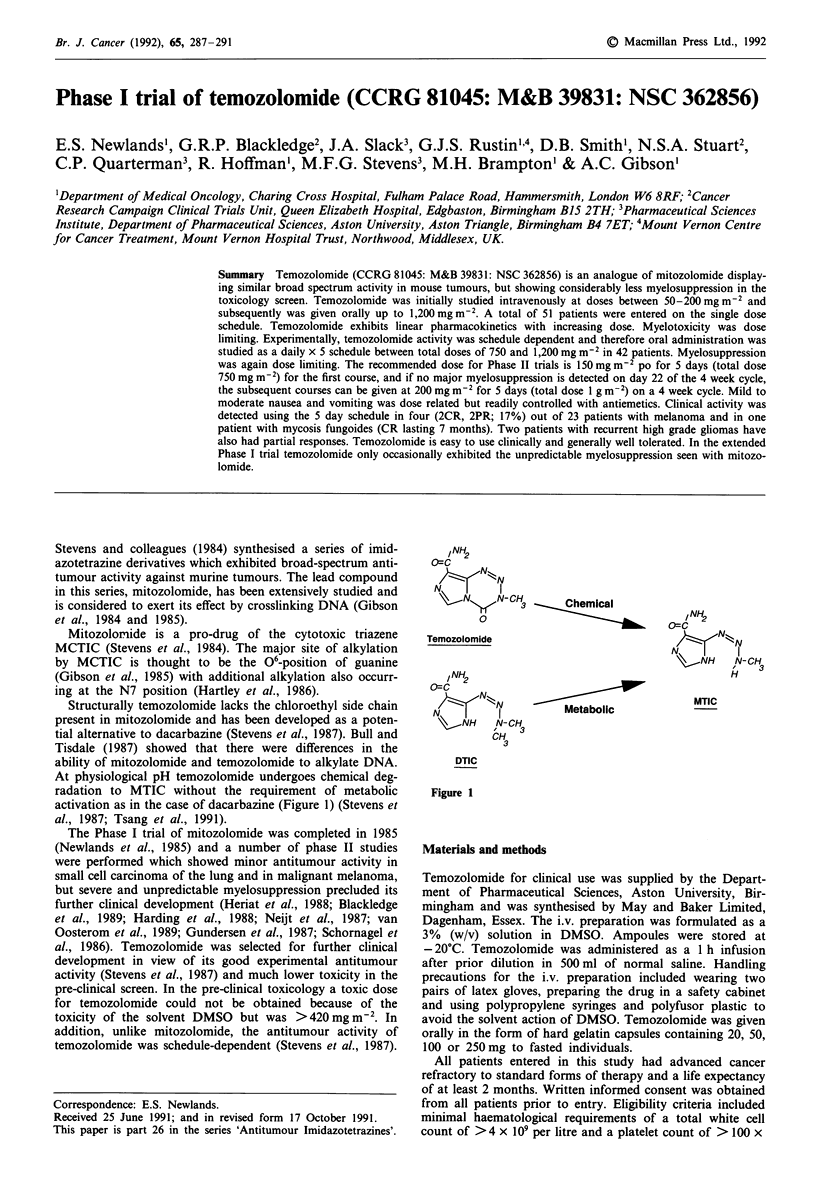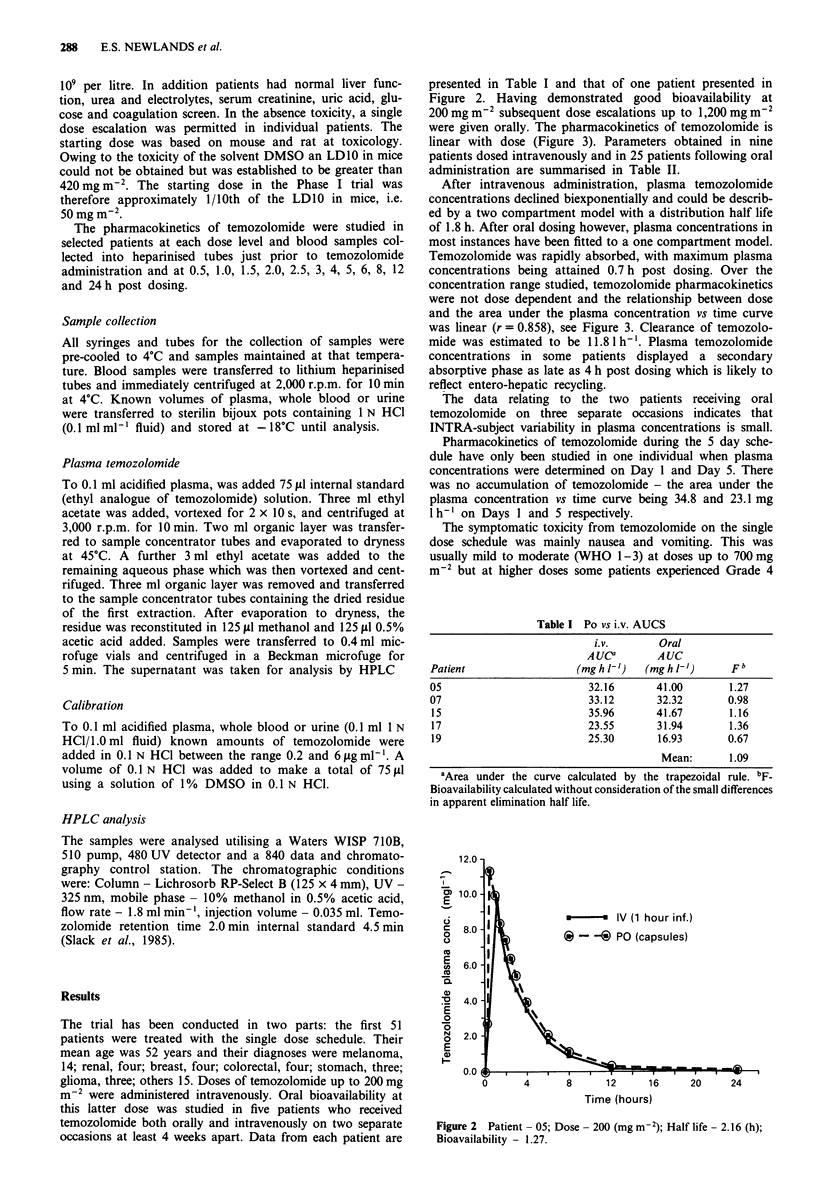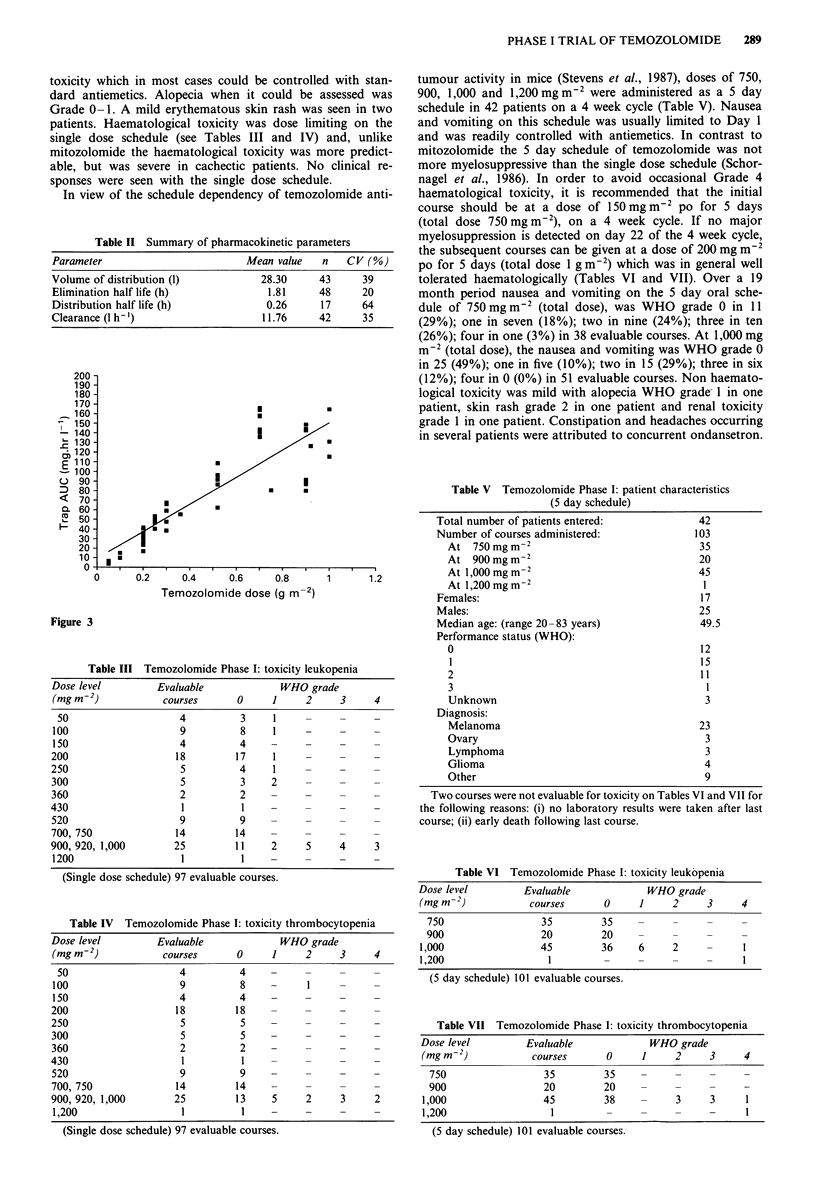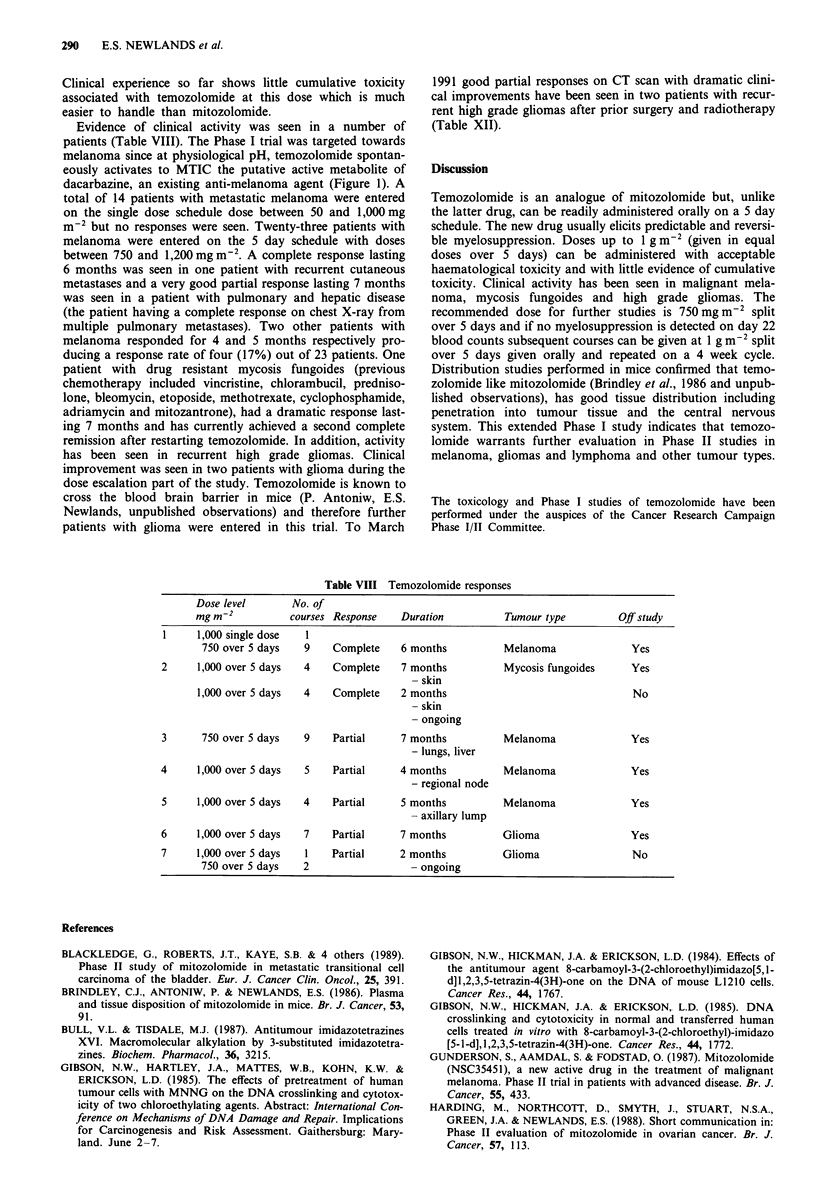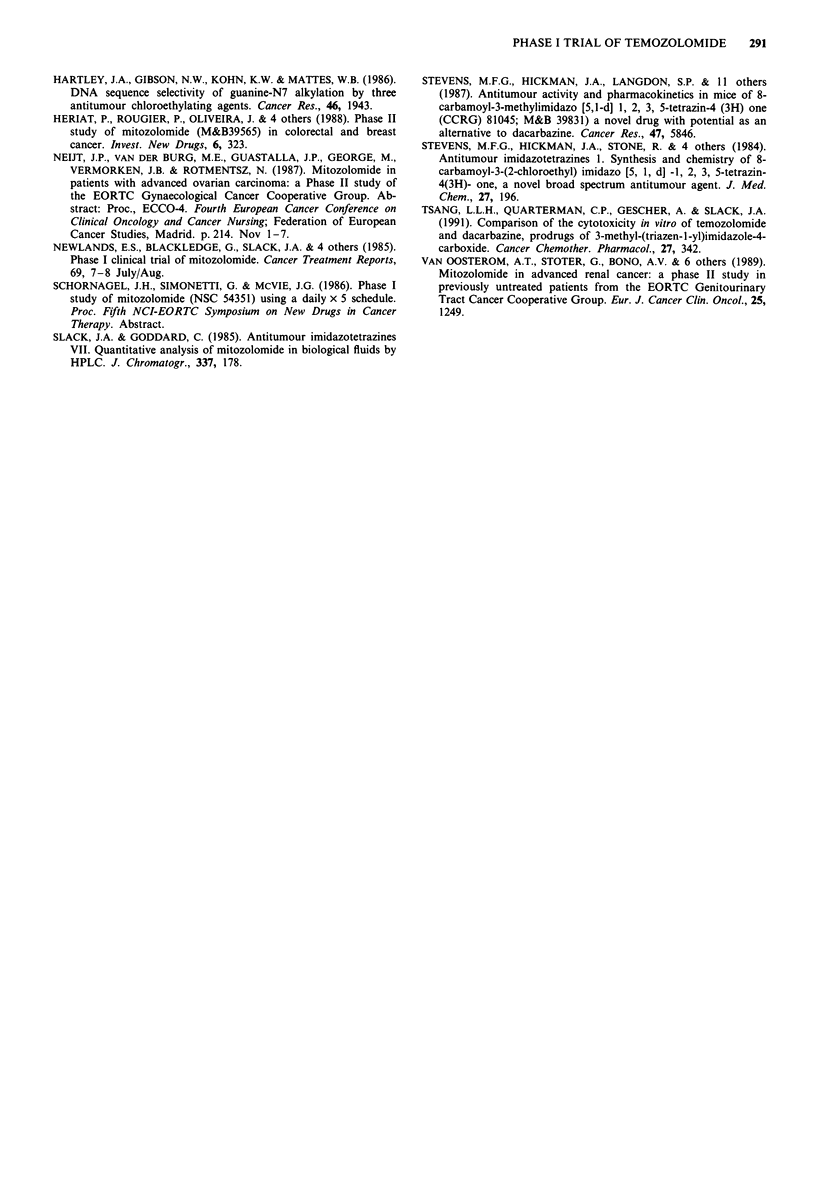# Phase I trial of temozolomide (CCRG 81045: M&B 39831: NSC 362856).

**DOI:** 10.1038/bjc.1992.57

**Published:** 1992-02

**Authors:** E. S. Newlands, G. R. Blackledge, J. A. Slack, G. J. Rustin, D. B. Smith, N. S. Stuart, C. P. Quarterman, R. Hoffman, M. F. Stevens, M. H. Brampton

**Affiliations:** Department of Medical Oncology, Charing Cross Hospital, Hammersmith, London, UK.

## Abstract

Temozolomide (CCRG 81045: M&B 39831: NSC 362856) is an analogue of mitozolomide displaying similar broad spectrum activity in mouse tumours, but showing considerably less myelosuppression in the toxicology screen. Temozolomide was initially studied intravenously at doses between 50-200 mg m-2 and subsequently was given orally up to 1,200 mg m-2. A total of 51 patients were entered on the single dose schedule. Temozolomide exhibits linear pharmacokinetics with increasing dose. Myelotoxicity was dose limiting. Experimentally, temozolomide activity was schedule dependent and therefore oral administration was studied as a daily x 5 schedule between total doses of 750 and 1,200 mg m-2 in 42 patients. Myelosuppression was again dose limiting. The recommended dose for Phase II trials is 150 mg m-2 po for 5 days (total dose 750 mg m-2) for the first course, and if no major myelosuppression is detected on day 22 of the 4 week cycle, the subsequent courses can be given at 200 mg m-2 for 5 days (total dose 1 g m-2) on a 4 week cycle. Mild to moderate nausea and vomiting was dose related but readily controlled with antiemetics. Clinical activity was detected using the 5 day schedule in four (2CR, 2PR; 17%) out of 23 patients with melanoma and in one patient with mycosis fungoides (CR lasting 7 months). Two patients with recurrent high grade gliomas have also had partial responses. Temozolomide is easy to use clinically and generally well tolerated. In the extended Phase I trial temozolomide only occasionally exhibited the unpredictable myelosuppression seen with mitozolomide.


*Br. J. Cancer* (1992), **65**, 287–291 © Macmillan Press Ltd., 1992

### Phase I trial of temozolomide (CCRG 81045: M&B 39831: NSC 362856)

E.S. Newlands[1], G.R.P. Blackledge[2], J.A. Slack[3], G.J.S. Rustin[1,4], D.B. Smith[1], N.S.A. Stuart[2], C.P. Quarterman[3], R. Hoffman[1], M.F.G. Stevens[3], M.H. Brampton[1] & A.C. Gibson[1]

[1]*Department of Medical Oncology, Charing Cross Hospital, Fulham Palace Road, Hammersmith, London W6 8RF;* [2]*Cancer Research Campaign Clinical Trials Unit, Queen Elizabeth Hospital, Edgbaston, Birmingham B15 2TH;* [3]*Pharmaceutical Sciences Institute, Department of Pharmaceutical Sciences, Aston University, Aston Triangle, Birmingham B4 7ET;* [4]*Mount Vernon Centre for Cancer Treatment, Mount Vernon Hospital Trust, Northwood, Middlesex, UK.*

**Summary** Temozolomide (CCRG 81045: M&B 39831: NSC 362856) is an analogue of mitozolomide displaying similar broad spectrum activity in mouse tumours, but showing considerably less myelosuppression in the toxicology screen. Temozolomide was initially studied intravenously at doses between 50–200 mg m$^{-2}$ and subsequently was given orally up to 1,200 mg m$^{-2}$. A total of 51 patients were entered on the single dose schedule. Temozolomide exhibits linear pharmacokinetics with increasing dose. Myelotoxicity was dose limiting. Experimentally, temozolomide activity was schedule dependent and therefore oral administration was studied as a daily × 5 schedule between total doses of 750 and 1,200 mg m$^{-2}$ in 42 patients. Myelosuppression was again dose limiting. The recommended dose for Phase II trials is 150 mg m$^{-2}$ po for 5 days (total dose 750 mg m$^{-2}$) for the first course, and if no major myelosuppression is detected on day 22 of the 4 week cycle, the subsequent courses can be given at 200 mg m$^{-2}$ for 5 days (total dose 1 g m$^{-2}$) on a 4 week cycle. Mild to moderate nausea and vomiting was dose related but readily controlled with antiemetics. Clinical activity was detected using the 5 day schedule in four (2CR, 2PR; 17%) out of 23 patients with melanoma and in one patient with mycosis fungoides (CR lasting 7 months). Two patients with recurrent high grade gliomas have also had partial responses. Temozolomide is easy to use clinically and generally well tolerated. In the extended Phase I trial temozolomide only occasionally exhibited the unpredictable myelosuppression seen with mitozolomide.

Stevens and colleagues (1984) synthesised a series of imidazotetrazine derivatives which exhibited broad-spectrum antitumour activity against murine tumours. The lead compound in this series, mitozolomide, has been extensively studied and is considered to exert its effect by crosslinking DNA (Gibson *et al.*, 1984 and 1985).

Mitozolomide is a pro-drug of the cytotoxic triazene MCTIC (Stevens *et al.*, 1984). The major site of alkylation by MCTIC is thought to be the O$^6$-position of guanine (Gibson *et al.*, 1985) with additional alkylation also occurring at the N7 position (Hartley *et al.*, 1986).

Structurally temozolomide lacks the chloroethyl side chain present in mitozolomide and has been developed as a potential alternative to dacarbazine (Stevens *et al.*, 1987). Bull and Tisdale (1987) showed that there were differences in the ability of mitozolomide and temozolomide to alkylate DNA. At physiological pH temozolomide undergoes chemical degradation to MTIC without the requirement of metabolic activation as in the case of dacarbazine (Figure 1) (Stevens *et al.*, 1987; Tsang *et al.*, 1991).

The Phase I trial of mitozolomide was completed in 1985 (Newlands *et al.*, 1985) and a number of phase II studies were performed which showed minor antitumour activity in small cell carcinoma of the lung and in malignant melanoma, but severe and unpredictable myelosuppression precluded its further clinical development (Heriat *et al.*, 1988; Blackledge *et al.*, 1989; Harding *et al.*, 1988; Neijt *et al.*, 1987; van Oosterom *et al.*, 1989; Gundersen *et al.*, 1987; Schornagel *et al.*, 1986). Temozolomide was selected for further clinical development in view of its good experimental antitumour activity (Stevens *et al.*, 1987) and much lower toxicity in the pre-clinical screen. In the pre-clinical toxicology a toxic dose for temozolomide could not be obtained because of the toxicity of the solvent DMSO but was >420 mg m$^{-2}$. In addition, unlike mitozolomide, the antitumour activity of temozolomide was schedule-dependent (Stevens *et al.*, 1987).

Correspondence: E.S. Newlands.
Received 25 June 1991; and in revised form 17 October 1991.
This paper is part 26 in the series 'Antitumour Imidazotetrazines'.

**Figure 1**

### Materials and methods

Temozolomide for clinical use was supplied by the Department of Pharmaceutical Sciences, Aston University, Birmingham and was synthesised by May and Baker Limited, Dagenham, Essex. The i.v. preparation was formulated as a 3% (w/v) solution in DMSO. Ampoules were stored at −20°C. Temozolomide was administered as a 1 h infusion after prior dilution in 500 ml of normal saline. Handling precautions for the i.v. preparation included wearing two pairs of latex gloves, preparing the drug in a safety cabinet and using polypropylene syringes and polyfusor plastic to avoid the solvent action of DMSO. Temozolomide was given orally in the form of hard gelatin capsules containing 20, 50, 100 or 250 mg to fasted individuals.

All patients entered in this study had advanced cancer refractory to standard forms of therapy and a life expectancy of at least 2 months. Written informed consent was obtained from all patients prior to entry. Eligibility criteria included minimal haematological requirements of a total white cell count of $>4 \times 10^9$ per litre and a platelet count of $>100 \times$

$10^9$ per litre. In addition patients had normal liver function, urea and electrolytes, serum creatinine, uric acid, glucose and coagulation screen. In the absence toxicity, a single dose escalation was permitted in individual patients. The starting dose was based on mouse and rat at toxicology. Owing to the toxicity of the solvent DMSO an LD10 in mice could not be obtained but was established to be greater than 420 mg $m^{-2}$. The starting dose in the Phase I trial was therefore approximately 1/10th of the LD10 in mice, i.e. 50 mg $m^{-2}$.

The pharmacokinetics of temozolomide were studied in selected patients at each dose level and blood samples collected into heparinised tubes just prior to temozolomide administration and at 0.5, 1.0, 1.5, 2.0, 2.5, 3, 4, 5, 6, 8, 12 and 24 h post dosing.

*Sample collection*

All syringes and tubes for the collection of samples were pre-cooled to 4°C and samples maintained at that temperature. Blood samples were transferred to lithium heparinised tubes and immediately centrifuged at 2,000 r.p.m. for 10 min at 4°C. Known volumes of plasma, whole blood or urine were transferred to sterilin bijoux pots containing 1 N HCl (0.1 ml $ml^{-1}$ fluid) and stored at −18°C until analysis.

*Plasma temozolomide*

To 0.1 ml acidified plasma, was added 75 μl internal standard (ethyl analogue of temozolomide) solution. Three ml ethyl acetate was added, vortexed for 2 × 10 s, and centrifuged at 3,000 r.p.m. for 10 min. Two ml organic layer was transferred to sample concentrator tubes and evaporated to dryness at 45°C. A further 3 ml ethyl acetate was added to the remaining aqueous phase which was then vortexed and centrifuged. Three ml organic layer was removed and transferred to the sample concentrator tubes containing the dried residue of the first extraction. After evaporation to dryness, the residue was reconstituted in 125 μl methanol and 125 μl 0.5% acetic acid added. Samples were transferred to 0.4 ml microfuge vials and centrifuged in a Beckman microfuge for 5 min. The supernatant was taken for analysis by HPLC

*Calibration*

To 0.1 ml acidified plasma, whole blood or urine (0.1 ml 1 N HCl/1.0 ml fluid) known amounts of temozolomide were added in 0.1 N HCl between the range 0.2 and 6 μg $ml^{-1}$. A volume of 0.1 N HCl was added to make a total of 75 μl using a solution of 1% DMSO in 0.1 N HCl.

*HPLC analysis*

The samples were analysed utilising a Waters WISP 710B, 510 pump, 480 UV detector and a 840 data and chromatography control station. The chromatographic conditions were: Column – Lichrosorb RP-Select B (125 × 4 mm), UV – 325 nm, mobile phase – 10% methanol in 0.5% acetic acid, flow rate – 1.8 ml $min^{-1}$, injection volume – 0.035 ml. Temozolomide retention time 2.0 min internal standard 4.5 min (Slack *et al.*, 1985).

### Results

The trial has been conducted in two parts: the first 51 patients were treated with the single dose schedule. Their mean age was 52 years and their diagnoses were melanoma, 14; renal, four; breast, four; colorectal, four; stomach, three; glioma, three; others 15. Doses of temozolomide up to 200 mg $m^{-2}$ were administered intravenously. Oral bioavailability at this latter dose was studied in five patients who received temozolomide both orally and intravenously on two separate occasions at least 4 weeks apart. Data from each patient are presented in Table I and that of one patient presented in Figure 2. Having demonstrated good bioavailability at 200 mg $m^{-2}$ subsequent dose escalations up to 1,200 mg $m^{-2}$ were given orally. The pharmacokinetics of temozolomide is linear with dose (Figure 3). Parameters obtained in nine patients dosed intravenously and in 25 patients following oral administration are summarised in Table II.

After intravenous administration, plasma temozolomide concentrations declined biexponentially and could be described by a two compartment model with a distribution half life of 1.8 h. After oral dosing however, plasma concentrations in most instances have been fitted to a one compartment model. Temozolomide was rapidly absorbed, with maximum plasma concentrations being attained 0.7 h post dosing. Over the concentration range studied, temozolomide pharmacokinetics were not dose dependent and the relationship between dose and the area under the plasma concentration *vs* time curve was linear ($r = 0.858$), see Figure 3. Clearance of temozolomide was estimated to be 11.8 l $h^{-1}$. Plasma temozolomide concentrations in some patients displayed a secondary absorptive phase as late as 4 h post dosing which is likely to reflect entero-hepatic recycling.

The data relating to the two patients receiving oral temozolomide on three separate occasions indicates that INTRA-subject variability in plasma concentrations is small.

Pharmacokinetics of temozolomide during the 5 day schedule have only been studied in one individual when plasma concentrations were determined on Day 1 and Day 5. There was no accumulation of temozolomide – the area under the plasma concentration *vs* time curve being 34.8 and 23.1 mg l $h^{-1}$ on Days 1 and 5 respectively.

The symptomatic toxicity from temozolomide on the single dose schedule was mainly nausea and vomiting. This was usually mild to moderate (WHO 1–3) at doses up to 700 mg $m^{-2}$ but at higher doses some patients experienced Grade 4

**Table I** Po *vs* i.v. AUCS

| *Patient* | *i.v. AUC*[a] *(mg h l⁻¹)* | *Oral AUC (mg h l⁻¹)* | *F*[b] |
|---|---|---|---|
| 05 | 32.16 | 41.00 | 1.27 |
| 07 | 33.12 | 32.32 | 0.98 |
| 15 | 35.96 | 41.67 | 1.16 |
| 17 | 23.55 | 31.94 | 1.36 |
| 19 | 25.30 | 16.93 | 0.67 |
| | | Mean: | 1.09 |

[a]Area under the curve calculated by the trapezoidal rule. [b]F-Bioavailability calculated without consideration of the small differences in apparent elimination half life.

**Figure 2** Patient – 05; Dose – 200 (mg $m^{-2}$); Half life – 2.16 (h); Bioavailability – 1.27.

toxicity which in most cases could be controlled with standard antiemetics. Alopecia when it could be assessed was Grade 0–1. A mild erythematous skin rash was seen in two patients. Haematological toxicity was dose limiting on the single dose schedule (see Tables III and IV) and, unlike mitozolomide the haematological toxicity was more predictable, but was severe in cachectic patients. No clinical responses were seen with the single dose schedule.

In view of the schedule dependency of temozolomide antitumour activity in mice (Stevens *et al.*, 1987), doses of 750, 900, 1,000 and 1,200 mg $m^{-2}$ were administered as a 5 day schedule in 42 patients on a 4 week cycle (Table V). Nausea and vomiting on this schedule was usually limited to Day 1 and was readily controlled with antiemetics. In contrast to mitozolomide the 5 day schedule of temozolomide was not more myelosuppressive than the single dose schedule (Schornagel *et al.*, 1986). In order to avoid occasional Grade 4 haematological toxicity, it is recommended that the initial course should be at a dose of 150 mg $m^{-2}$ po for 5 days (total dose 750 mg $m^{-2}$), on a 4 week cycle. If no major myelosuppression is detected on day 22 of the 4 week cycle, the subsequent courses can be given at a dose of 200 mg $m^{-2}$ po for 5 days (total dose 1 g $m^{-2}$) which was in general well tolerated haematologically (Tables VI and VII). Over a 19 month period nausea and vomiting on the 5 day oral schedule of 750 mg $m^{-2}$ (total dose), was WHO grade 0 in 11 (29%); one in seven (18%); two in nine (24%); three in ten (26%); four in one (3%) in 38 evaluable courses. At 1,000 mg $m^{-2}$ (total dose), the nausea and vomiting was WHO grade 0 in 25 (49%); one in five (10%); two in 15 (29%); three in six (12%); four in 0 (0%) in 51 evaluable courses. Non haematological toxicity was mild with alopecia WHO grade 1 in one patient, skin rash grade 2 in one patient and renal toxicity grade 1 in one patient. Constipation and headaches occurring in several patients were attributed to concurrent ondansetron.

**Table II** Summary of pharmacokinetic parameters

| *Parameter* | *Mean value* | *n* | *CV (%)* |
|---|---|---|---|
| Volume of distribution (l) | 28.30 | 43 | 39 |
| Elimination half life (h) | 1.81 | 48 | 20 |
| Distribution half life (h) | 0.26 | 17 | 64 |
| Clearance (l $h^{-1}$) | 11.76 | 42 | 35 |

**Figure 3**

**Table III** Temozolomide Phase I: toxicity leukopenia

| *Dose level (mg $m^{-2}$)* | *Evaluable courses* | *WHO grade* 0 | 1 | 2 | 3 | 4 |
|---|---|---|---|---|---|---|
| 50 | 4 | 3 | 1 | – | – | – |
| 100 | 9 | 8 | 1 | – | – | – |
| 150 | 4 | 4 | – | – | – | – |
| 200 | 18 | 17 | 1 | – | – | – |
| 250 | 5 | 4 | 1 | – | – | – |
| 300 | 5 | 3 | 2 | – | – | – |
| 360 | 2 | 2 | – | – | – | – |
| 430 | 1 | 1 | – | – | – | – |
| 520 | 9 | 9 | – | – | – | – |
| 700, 750 | 14 | 14 | – | – | – | – |
| 900, 920, 1,000 | 25 | 11 | 2 | 5 | 4 | 3 |
| 1200 | 1 | 1 | – | – | – | – |

(Single dose schedule) 97 evaluable courses.

**Table IV** Temozolomide Phase I: toxicity thrombocytopenia

| *Dose level (mg $m^{-2}$)* | *Evaluable courses* | *WHO grade* 0 | 1 | 2 | 3 | 4 |
|---|---|---|---|---|---|---|
| 50 | 4 | 4 | – | – | – | – |
| 100 | 9 | 8 | – | 1 | – | – |
| 150 | 4 | 4 | – | – | – | – |
| 200 | 18 | 18 | – | – | – | – |
| 250 | 5 | 5 | – | – | – | – |
| 300 | 5 | 5 | – | – | – | – |
| 360 | 2 | 2 | – | – | – | – |
| 430 | 1 | 1 | – | – | – | – |
| 520 | 9 | 9 | – | – | – | – |
| 700, 750 | 14 | 14 | – | – | – | – |
| 900, 920, 1,000 | 25 | 13 | 5 | 2 | 3 | 2 |
| 1,200 | 1 | 1 | – | – | – | – |

(Single dose schedule) 97 evaluable courses.

**Table V** Temozolomide Phase I: patient characteristics (5 day schedule)

| | |
|---|---|
| Total number of patients entered: | 42 |
| Number of courses administered: | 103 |
| At 750 mg $m^{-2}$ | 35 |
| At 900 mg $m^{-2}$ | 20 |
| At 1,000 mg $m^{-2}$ | 45 |
| At 1,200 mg $m^{-2}$ | 1 |
| Females: | 17 |
| Males: | 25 |
| Median age: (range 20–83 years) | 49.5 |
| Performance status (WHO): | |
| 0 | 12 |
| 1 | 15 |
| 2 | 11 |
| 3 | 1 |
| Unknown | 3 |
| Diagnosis: | |
| Melanoma | 23 |
| Ovary | 3 |
| Lymphoma | 3 |
| Glioma | 4 |
| Other | 9 |

Two courses were not evaluable for toxicity on Tables VI and VII for the following reasons: (i) no laboratory results were taken after last course; (ii) early death following last course.

**Table VI** Temozolomide Phase I: toxicity leukopenia

| *Dose level (mg $m^{-2}$)* | *Evaluable courses* | *WHO grade* 0 | 1 | 2 | 3 | 4 |
|---|---|---|---|---|---|---|
| 750 | 35 | 35 | – | – | – | – |
| 900 | 20 | 20 | – | – | – | – |
| 1,000 | 45 | 36 | 6 | 2 | – | 1 |
| 1,200 | 1 | – | – | – | – | 1 |

(5 day schedule) 101 evaluable courses.

**Table VII** Temozolomide Phase I: toxicity thrombocytopenia

| *Dose level (mg $m^{-2}$)* | *Evaluable courses* | *WHO grade* 0 | 1 | 2 | 3 | 4 |
|---|---|---|---|---|---|---|
| 750 | 35 | 35 | – | – | – | – |
| 900 | 20 | 20 | – | – | – | – |
| 1,000 | 45 | 38 | – | 3 | 3 | 1 |
| 1,200 | 1 | – | – | – | – | 1 |

(5 day schedule) 101 evaluable courses.

Clinical experience so far shows little cumulative toxicity associated with temozolomide at this dose which is much easier to handle than mitozolomide.

Evidence of clinical activity was seen in a number of patients (Table VIII). The Phase I trial was targeted towards melanoma since at physiological pH, temozolomide spontaneously activates to MTIC the putative active metabolite of dacarbazine, an existing anti-melanoma agent (Figure 1). A total of 14 patients with metastatic melanoma were entered on the single dose schedule dose between 50 and 1,000 mg $m^{-2}$ but no responses were seen. Twenty-three patients with melanoma were entered on the 5 day schedule with doses between 750 and 1,200 mg $m^{-2}$. A complete response lasting 6 months was seen in one patient with recurrent cutaneous metastases and a very good partial response lasting 7 months was seen in a patient with pulmonary and hepatic disease (the patient having a complete response on chest X-ray from multiple pulmonary metastases). Two other patients with melanoma responded for 4 and 5 months respectively producing a response rate of four (17%) out of 23 patients. One patient with drug resistant mycosis fungoides (previous chemotherapy included vincristine, chlorambucil, prednisolone, bleomycin, etoposide, methotrexate, cyclophosphamide, adriamycin and mitozantrone), had a dramatic response lasting 7 months and has currently achieved a second complete remission after restarting temozolomide. In addition, activity has been seen in recurrent high grade gliomas. Clinical improvement was seen in two patients with glioma during the dose escalation part of the study. Temozolomide is known to cross the blood brain barrier in mice (P. Antoniw, E.S. Newlands, unpublished observations) and therefore further patients with glioma were entered in this trial. To March 1991 good partial responses on CT scan with dramatic clinical improvements have been seen in two patients with recurrent high grade gliomas after prior surgery and radiotherapy (Table XII).

### Discussion

Temozolomide is an analogue of mitozolomide but, unlike the latter drug, can be readily administered orally on a 5 day schedule. The new drug usually elicits predictable and reversible myelosuppression. Doses up to 1 g $m^{-2}$ (given in equal doses over 5 days) can be administered with acceptable haematological toxicity and with little evidence of cumulative toxicity. Clinical activity has been seen in malignant melanoma, mycosis fungoides and high grade gliomas. The recommended dose for further studies is 750 mg $m^{-2}$ split over 5 days and if no myelosuppression is detected on day 22 blood counts subsequent courses can be given at 1 g $m^{-2}$ split over 5 days given orally and repeated on a 4 week cycle. Distribution studies performed in mice confirmed that temozolomide like mitozolomide (Brindley *et al.*, 1986 and unpublished observations), has good tissue distribution including penetration into tumour tissue and the central nervous system. This extended Phase I study indicates that temozolomide warrants further evaluation in Phase II studies in melanoma, gliomas and lymphoma and other tumour types.

The toxicology and Phase I studies of temozolomide have been performed under the auspices of the Cancer Research Campaign Phase I/II Committee.

**Table VIII** Temozolomide responses

| | *Dose level mg $m^{-2}$* | *No. of courses* | *Response* | *Duration* | *Tumour type* | *Off study* |
|---|---|---|---|---|---|---|
| 1 | 1,000 single dose<br>750 over 5 days | 1<br>9 | Complete | 6 months | Melanoma | Yes |
| 2 | 1,000 over 5 days | 4 | Complete | 7 months<br>– skin | Mycosis fungoides | Yes |
| | 1,000 over 5 days | 4 | Complete | 2 months<br>– skin<br>– ongoing | | No |
| 3 | 750 over 5 days | 9 | Partial | 7 months<br>– lungs, liver | Melanoma | Yes |
| 4 | 1,000 over 5 days | 5 | Partial | 4 months<br>– regional node | Melanoma | Yes |
| 5 | 1,000 over 5 days | 4 | Partial | 5 months<br>– axillary lump | Melanoma | Yes |
| 6 | 1,000 over 5 days | 7 | Partial | 7 months | Glioma | Yes |
| 7 | 1,000 over 5 days<br>750 over 5 days | 1<br>2 | Partial | 2 months<br>– ongoing | Glioma | No |

### References

BLACKLEDGE, G., ROBERTS, J.T., KAYE, S.B. & 4 others (1989). Phase II study of mitozolomide in metastatic transitional cell carcinoma of the bladder. *Eur. J. Cancer Clin. Oncol.*, **25**, 391.

BRINDLEY, C.J., ANTONIW, P. & NEWLANDS, E.S. (1986). Plasma and tissue disposition of mitozolomide in mice. *Br. J. Cancer*, **53**, 91.

BULL, V.L. & TISDALE, M.J. (1987). Antitumour imidazotetrazines XVI. Macromolecular alkylation by 3-substituted imidazotetrazines. *Biochem. Pharmacol.*, **36**, 3215.

GIBSON, N.W., HARTLEY, J.A., MATTES, W.B., KOHN, K.W. & ERICKSON, L.D. (1985). The effects of pretreatment of human tumour cells with MNNG on the DNA crosslinking and cytotoxicity of two chloroethylating agents. Abstract: *International Conference on Mechanisms of DNA Damage and Repair.* Implications for Carcinogenesis and Risk Assessment. Gaithersburg: Maryland. June 2–7.

GIBSON, N.W., HICKMAN, J.A. & ERICKSON, L.D. (1984). Effects of the antitumour agent 8-carbamoyl-3-(2-chloroethyl)imidazo[5,1-d]1,2,3,5-tetrazin-4(3H)-one on the DNA of mouse L1210 cells. *Cancer Res.*, **44**, 1767.

GIBSON, N.W., HICKMAN, J.A. & ERICKSON, L.D. (1985). DNA crosslinking and cytotoxicity in normal and transferred human cells treated *in vitro* with 8-carbamoyl-3-(2-chloroethyl)-imidazo [5-1-d],1,2,3,5-tetrazin-4(3H)-one. *Cancer Res.*, **44**, 1772.

GUNDERSON, S., AAMDAL, S. & FODSTAD, O. (1987). Mitozolomide (NSC35451), a new active drug in the treatment of malignant melanoma. Phase II trial in patients with advanced disease. *Br. J. Cancer*, **55**, 433.

HARDING, M., NORTHCOTT, D., SMYTH, J., STUART, N.S.A., GREEN, J.A. & NEWLANDS, E.S. (1988). Short communication in: Phase II evaluation of mitozolomide in ovarian cancer. *Br. J. Cancer*, **57**, 113.

HARTLEY, J.A., GIBSON, N.W., KOHN, K.W. & MATTES, W.B. (1986). DNA sequence selectivity of guanine-N7 alkylation by three antitumour chloroethylating agents. *Cancer Res.*, **46**, 1943.

HERIAT, P., ROUGIER, P., OLIVEIRA, J. & 4 others (1988). Phase II study of mitozolomide (M&B39565) in colorectal and breast cancer. *Invest. New Drugs*, **6**, 323.

NEIJT, J.P., VAN DER BURG, M.E., GUASTALLA, J.P., GEORGE, M., VERMORKEN, J.B. & ROTMENTSZ, N. (1987). Mitozolomide in patients with advanced ovarian carcinoma: a Phase II study of the EORTC Gynaecological Cancer Cooperative Group. Abstract: Proc., ECCO-4. *Fourth European Cancer Conference on Clinical Oncology and Cancer Nursing*; Federation of European Cancer Studies, Madrid. p. 214. Nov 1–7.

NEWLANDS, E.S., BLACKLEDGE, G., SLACK, J.A. & 4 others (1985). Phase I clinical trial of mitozolomide. *Cancer Treatment Reports*, 69, 7–8 July/Aug.

SCHORNAGEL, J.H., SIMONETTI, G. & MCVIE, J.G. (1986). Phase I study of mitozolomide (NSC 54351) using a daily × 5 schedule. *Proc. Fifth NCI-EORTC Symposium on New Drugs in Cancer Therapy*. Abstract.

SLACK, J.A. & GODDARD, C. (1985). Antitumour imidazotetrazines VII. Quantitative analysis of mitozolomide in biological fluids by HPLC. *J. Chromatogr.*, **337**, 178.

STEVENS, M.F.G., HICKMAN, J.A., LANGDON, S.P. & 11 others (1987). Antitumour activity and pharmacokinetics in mice of 8-carbamoyl-3-methylimidazo [5,1-d] 1, 2, 3, 5-tetrazin-4 (3H) one (CCRG) 81045; M&B 39831) a novel drug with potential as an alternative to dacarbazine. *Cancer Res.*, **47**, 5846.

STEVENS, M.F.G., HICKMAN, J.A., STONE, R. & 4 others (1984). Antitumour imidazotetrazines 1. Synthesis and chemistry of 8-carbamoyl-3-(2-chloroethyl) imidazo [5, 1, d] -1, 2, 3, 5-tetrazin-4(3H)- one, a novel broad spectrum antitumour agent. *J. Med. Chem.*, **27**, 196.

TSANG, L.L.H., QUARTERMAN, C.P., GESCHER, A. & SLACK, J.A. (1991). Comparison of the cytotoxicity *in vitro* of temozolomide and dacarbazine, prodrugs of 3-methyl-(triazen-1-yl)imidazole-4-carboxide. *Cancer Chemother. Pharmacol.*, **27**, 342.

VAN OOSTEROM, A.T., STOTER, G., BONO, A.V. & 6 others (1989). Mitozolomide in advanced renal cancer: a phase II study in previously untreated patients from the EORTC Genitourinary Tract Cancer Cooperative Group. *Eur. J. Cancer Clin. Oncol.*, **25**, 1249.